# Knowledge, attitudes, and practices of vaccinators about expanded programs on immunization: a cross-sectional study

**DOI:** 10.3389/fpubh.2024.1366378

**Published:** 2024-03-06

**Authors:** Sunia Azhar, Laiba Rashid, Taskeen Islam, Samar Akhtar, Kathryn L. Hopkins, Theresa Sommers, Aamer Ikram, Naveed Anwer, Nabeel Ahmed Maqbool, Zakir Khan, Naveed Ahmed, Hashaam Akhtar

**Affiliations:** ^1^Yusra Institute of Pharmaceutical Sciences, Islamabad, Pakistan; ^2^Communication and Media Studies, Fatima Jinnah Women University, Rawalpindi, Pakistan; ^3^Yusra Institute of Pharmaceutical Sciences, Yusra Medical and Dental College, Zaraj Housing Society, Islamabad, Pakistan; ^4^Sabin Vaccine Institute, Washington, DC, United States; ^5^Armed Forces Institute of Pathology, Rawalpindi, Pakistan; ^6^Department of Pharmacy, Quaid.i.Azam University, Islamabad, Pakistan; ^7^Vaccines Preventable Infectious Diseases, Chemonics International Global Health Supply Chain – Procurement and Supply, Management (GHSC-PSM) Project, Islamabad, Pakistan; ^8^Department of Pharmacy Practice, Riphah Institute of Pharmaceutical Sciences (RIPS), Riphah International University Gulberg Green Campus, Islamabad, Pakistan; ^9^Department of Global Health, Health Services Academy, Chak Shahzad, Islamabad, Pakistan

**Keywords:** vaccines, immunization, health, knowledge, attitude, and practice (KAP), Pakistan

## Abstract

**Introduction:**

The periodic evaluation of knowledge, attitudes, and practices (KAP) of healthcare workers, including vaccinators, concerning expanded programs on immunization (EPI) is very crucial for a better healthcare system. This study was carried out to assess the KAP of vaccinators about the EPI, including cold storage of vaccines and their practices related to vaccine cold chain management.

**Method:**

A cross-sectional study was conducted from January 2022 to June 2022 among registered vaccinators in the twin cities (Islamabad and Rawalpindi) of Pakistan. A structured self-administered questionnaire (English and Urdu) was developed as per the Pakistan national EPI policy and strategic guidelines 2022 and World Health Organization (WHO) guidelines, as well as from earlier studies (Cronbach's alpha value of 0.734). The final questionnaire consisted of closed-ended questions in four sections, including sociodemographic information, knowledge (with dichotomous variables of yes/no), attitudes (with a 5-point Likert scale ranging from strongly agree to strongly disagree), and handling of vaccines and cold chain management. Completed questionnaires were entered into Microsoft Excel and then imported into SPSS version 25 for statistical analysis.

**Results:**

A total of 186 vaccinators completely filled out their questionnaires, with a 97.9% response rate. More than half of the participants (57.5%) had no training related to EPI. Most of the respondents had a moderate to poor level of knowledge regarding EPI. The overall attitude was positive, and 57% of the participants strongly agreed that the national immunization programs can significantly contribute to the decrease in morbidity and mortality rates among children. In the current study, participants showed good practices toward EPI, vaccine storage, and cold chain management. The majority (93.5%) of the participants checked the expiry of vaccines at regular intervals to maintain the first expiry first out (FEFO) in their healthcare setting.

**Discussion:**

In conclusion, most of the vaccinators had moderate to poor knowledge, a positive attitude, and good practices toward EPI, vaccine cold storage, and cold chain management. Lack of training among vaccinators on EPI was also observed. These findings have suggested that continuous training, education, and regular supervision of vaccinators in EPI are important for maximum immunization effectiveness and coverage.

## Background

The vaccine refers to a suspension of weakened, killed, or fragmented microorganisms, toxins, or other biological preparations, such as those consisting of antibodies, lymphocytes, or messenger RNA (mRNA), that is administered primarily to prevent diseases (1). Vaccination is one of the most powerful and cost-effective public health interventions that has the greatest impact on public health, saving millions of lives each year (2). Under-vaccination and non-vaccination are major problems in low- and middle-income countries (LMICs) (3). It is documented that Pakistan is the third most unvaccinated country in the world (4). Every year, almost three million children miss out on an entire course of the most readily available vaccines, leaving them vulnerable to life-threatening diseases (4, 5). Children under 5 years of age make up 15% of the population in Pakistan (4). Only two-thirds of these children receive all basic vaccinations, which lags behind the global average (85%). The country is currently home to two million unvaccinated children, and approximately half of the deaths among children under 5 years of age occur due to vaccine-preventable diseases (VPDs) (4). While evidence of the value and efficacy of vaccination is well established, there is still a gap in our knowledge and understanding of ways to improve the implementation, enhance effectiveness, and scale-up this life-saving intervention to identify and vaccinate zero-dose and under-vaccinated children, particularly among the most deprived populations (5).

The Extended Program on Immunization (EPI) was launched in 1974 by the World Health Organization (WHO) to control VPDs such as tuberculosis, diphtheria, pertussis, tetanus, polio, and measles (6, 7). The morbidity and mortality rates of VPDs tended to decline in many countries even after achieving high immunization coverage (8). The WHO recommends implementing the EPI global program in a country-specific manner to adjust the vaccination programs and strategies to the national interests and situation since each country's immunization needs and challenges differ (5). In 2012, 194 member states of the World Health Assembly endorsed the “Global Vaccine Action Plan” (GVAP), which laid out instructions that are both for global and country-specific perspectives (9).

In Pakistan, the EPI is implemented at the federal level by the Ministry of National Health Services, Regulation, and Coordination (NHSRC) and at the provincial level by the provincial health departments. Various stakeholders (district health authorities, private sector providers, lady health workers, civil society organizations, media, and communities) and development partners (WHO, UNICEF, USAID, the World Bank, Rotary International, and the Bill & Melinda Gates Foundation) are involved in the EPI to provide technical support and financial assistance (10). In 1978, Pakistan launched its EPI program, and with the assistance of development partners, numerous other vaccinations were introduced to the EPI between 2001 and 2015, including hepatitis B, haemophilus influenza type B (HiB), pneumococcal vaccine (PCV 10), and inactivated polio vaccine (5). However, Pakistan is still dealing with polio and has long been the focus of concern for polio eradication, alongside Afghanistan and Nigeria (11). It is significant to note that Pakistan is considered a “priority country” for immunization (5). Pakistan is one of the two remaining countries where wild poliovirus transmission still occurs. Wild poliovirus type 1 (WPV1) cases in Pakistan in 2019 were 147, 84 in 2020, and a single case was recorded in 2021; however, a total of 20 cases were reported in 2022, indicating that Pakistan is a high-priority country that poses a risk of poliovirus spread to other countries (12). Measles can be prevented by vaccination, but many children in Pakistan still neglect to get their scheduled immunizations. Pakistan reported 4,531 measles cases, which is the highest, and 351 diphtheria cases in 2020, which is the second highest in the WHO Eastern Mediterranean Region (13, 14).

In Pakistan, the maintenance of the vaccine cold chain is one of the major challenges in the implementation of the EPI. All healthcare workers are responsible for controlling several aspects of cold chain management, including handling, vaccine storage, administration, and transportation (15). Healthcare workers, including vaccinators, play a critical role in educating, guiding, and encouraging vaccinations based on the latest scientific research as a prophylactic measure for protecting oneself from the hazards of acquiring vaccine-preventable illnesses and, as a result, their spread to patients, the community, and members of high-risk and vulnerable groups (16, 17). Vaccinators also hold a pivotal responsibility in regulating the EPI, as they not only have the responsibility of communicating the knowledge on how to store vaccines effectively and how to effectively manage the cold chain, but they also effectively advocate for vaccinations based on the most relevant scientific proof (18, 19). This interaction between healthcare workers and the EPI highlights the crucial association between well-informed health caregivers and the broader goals of immunization programs (2).

The periodic evaluation of knowledge, attitudes, and practices (KAP) of vaccinators concerning the EPI is very important (18–20). However, to the best of our knowledge, there has been no published study about vaccinators' KAP regarding the EPI in Pakistan. Therefore, this study was carried out to assess the KAP of vaccinators about the EPI, including cold storage of vaccines and their practices related to vaccine cold chain management.

## Methodology

### Study design

A cross-sectional questionnaire-based study was carried out among registered vaccinators. The data collection for this study was conducted from January 2022 to June 2022. All vaccinators included in the study provided their consent to participate. All other people not related to the medical field and those who did not have a job description as vaccinators were excluded.

### Study place

Twin cities in Pakistan (Islamabad and Rawalpindi) were selected for the current study. Islamabad has two zones (the Capital Development Authority (CDA) zone and the rural zone) with 27 fixed vaccination sites while Rawalpindi has 212 union councils with 157 EPI fixed sites that provide vaccination health services. These sites were selected for data collection.

### Sample size

There are 110 registered vaccinators (CDA zone: 45 and rural zone: 65) in Islamabad and 226 vaccinators in Rawalpindi. The minimal sample size required was calculated using a single population proportion formula. There were 336 registered vaccinators during the study period. A minimum sample size of 180 was indicated using the Raosoft sample size calculator http://www.raosoft.com/samplesize.html. A 50% response distribution, a 5% error margin, and a 95% confidence level were assumed for the purpose of estimating the sample size. We distributed the questionnaires to 190 vaccinators to account for the response rate or any missing data.

### Data collection method

A structured self-administered questionnaire was developed as per the Pakistan national EPI policy and strategic guidelines 2022 and WHO guidelines (21), as well as from earlier studies (19, 22, 23). The questionnaire was prepared in both English and Urdu (the official language of Pakistan) for the ease of understanding. Three competent and experienced researchers skilled in reading and writing Urdu and English revised the questionnaire. Minor modifications were recommended after conducting face and content validity testing. The final instrument was then amended as per their recommendations. A pilot study was also conducted with 10 vaccinators. The pilot study was designed to evaluate the study tool's applicability and clarity as well as to identify any potential concerns that might develop during the data collection. The findings of the pilot study were satisfactory, and the reliability coefficient (Cronbach's alpha value) of 0.734 was calculated. Trained members of the study team gathered the information prospectively by distributing a face-to-face questionnaire. The goal of the study and data confidentiality were explained to the respondents, and informed consent (oral and written) on their willingness to participate was obtained.

### Data collection tool

The final questionnaire consisted of closed-ended questions in four sections. The first section included sociodemographic information (sex, age, educational background, years of service in EPI, and training on EPI), while the second and third sections consisted of knowledge and attitudes of vaccinators about EPI, respectively. The final part consisted of questions related to the handling of vaccines and cold chain management. Knowledge and practice were assessed using dichotomous (yes/no) variables, while attitudes were assessed using a 5-point Likert scale (ranging from strongly agree to strongly disagree) (Supplementary material 1).

### Data management and analysis

The final questionnaire was checked for any discrepancies and missing data. Fully completed questionnaires were entered into Microsoft Excel (Microsoft Corporation) and then imported into SPSS version 25 (Chicago, Illinois, U.S.A.) for statistical analysis. Descriptive statistics (mean, percentage, and frequency) were used to describe the characteristics of the study variables and sample population. Inferential statistical (chi-squared test) analysis was performed to assess the correlation between the demographic variables and KAP questions. The overall percent score cutoff was computed using the modified Bloom's cutoff point criteria, which had already been used in previous studies (24, 25). We chose nine questions for the knowledge part, with each accurate response (yes:1) receiving one point and the incorrect response (no:0) receiving zero points. The overall score for all items was nine. The vaccinators were defined as having “good knowledge” if their score was above 75% (ranging from 77.7% to 100% with 7–8 points), “moderate knowledge” if their score ranged from 55% to 74% (5–6 points), and “poor knowledge” if their score was < 55% (< 5 points). The total practice score was categorized as “good practice” if it ranged from 75% to 100% (9–12 points) or “poor practice” if it was < 75% (8 points) (24, 25). Logistic regression analysis was used to identify the differences in sociodemographic characteristics between training and non-training variables. *P*-values of < 0.05 were considered statistically significant.

### Ethics approval and consent to participate

This study was approved by the ethics committee of Yusra Institute of Pharmaceutical Sciences (Reference number: YIPS/4065/EC-17; Dated: 01/01/2021) (Supplementary material 2). Informed consent (both oral and written) was obtained from all participants. The goals of the study were explained to the participants to establish the significance of the research. Furthermore, the confidentiality of the participant's data was assured.

## Results

### Sociodemographic details

A total of 186 vaccinators completely filled out their questionnaires, with a 97.9% response rate. The current study includes a higher proportion of male participants (*n* = 123; 66.1%) compared to female participants. Most of the participants had a bachelor's degree (62.4%), followed by an intermediate (15.6%) and a master's degree (14.5%). Most of the respondents were new recruits and had no prior job experience (43.0%). Additionally, one-quarter (25.8%) of the participants had job experience of 1–2 years, and 15.60% of the participants had job experience of more than 5 years of service. More than half of the participants (57.5%) had no training related to EPI. The most common workplaces for vaccinators were private hospitals (37.1%), followed by public health hospitals (32.3%) and teaching academic hospitals (14.0%) (Table 1).

**Table 1:** Sociodemographic characteristics of participants (*n* = 186).

**Variable**	**Characteristics**	**Frequency**	**Percentage**
Sex	Female	123	66.1%
	Male	63	33.9%
Educational level	Matric	14	7.5%
	Intermediate	29	15.6%
	Bachelor's	116	62.4%
	Master's	27	14.5%
Years of service (years)	None	80	43.0%
	1–2	48	25.8%
	3–4	16	8.6%
	4–5	13	7.0 %
	More than 5	29	15.6%
Training	Yes	79	42.5%
	No	107	57.5%
Workplace	Teaching academic hospital	26	14.0 %
	Ministry of health	8	4.3%
	Public health hospital	60	32.3%
	Private hospital	69	37.1%
	Military hospital	23	12.4%

### Knowledge of vaccinators about EPI

Most of the healthcare workers had a moderate to poor level of knowledge regarding EPI. A majority of the participants (76.9%) responded correctly about the vaccination delay in persons with high-grade fever. Approximately two-thirds (62.4%) of the participants were aware that patients with chronic liver, kidney, or heart diseases can receive vaccinations. Over half (59.1%) of the participants responded correctly when inquired about diarrhea symptoms in children before administering the polio vaccine. Furthermore, 57% of the participants knew that immunocompromised individuals could not receive live vaccines. Approximately half of the vaccinators (49.5%) gave a correct response about the dosage adjustment of the oral polio vaccine (OPV) based on the weight of the neonatal. More than half (54.8%) of the participants responded correctly about the repetition of the pentavalent vaccine (DPT+HBV+HIB) despite its adverse effects. A statistically significant difference was observed among knowledge variables with training and non-training of vaccinators (*p*-value < 0.05) (Table 2).

**Table 2:** Knowledge of vaccinators regarding EPI.

**Questions**	**Corrected response (*n* = 186)**	**Training**		***p*-value**
	***n*** **(%)**	**Yes (**=**79)**	**No (*****n*** = **107)**	
Can BCG be given to HIV positive patients?	60 (32.2)	37 (46.8)	57 (53.3)	0.191
Do you ask for diarrhea symptoms of children before administering the polio vaccine?	110 (59.1)	54 (68.3)	56 (52.3)	**0.009**
Can we adjust the dose of OPV according to weight of neonates?	66 (35.5)	29 (36.7)	37 (34.5)	**0.001**
Can a dose of pentavalent (DPT+HBV+HIB) be repeated if adverse effects are reported?	64 (34.4)	30 (37.9)	34 (31.7)	0.596
Can live vaccines be given to immunocompromised individuals?	69 (37.1)	38 (48.1)	31 (28.9)	**0.022**
Is it necessary to administer multiple doses of the same antigen to an individual after 4 weeks of the first dose?	105 (56.4)	44 (55.7)	61 (57.0)	0.552
Is it recommended to delay vaccination in persons having high-grade fever >39°C?	143 (76.9)	60 (75.9)	83 (77.5)	0.853
Can persons having chronic kidney, liver, or heart diseases receive vaccination?	116 (62.4)	68 (86.0)	48 (44.8)	0.713
Can patients on medications (such as antibiotics or corticosteroids) receive vaccination?	105 (56.5)	62 (78.5)	43 (40.1)	**0.018**

Bold values indicate the *p* < 0.05.

### Attitudes of vaccinators regarding EPI

In this study, the overall attitude of vaccinators about EPI was positive, and 57.0% of the participants strongly agreed that the national immunization programs can significantly contribute to the decrease in morbidity and mortality rates among children. More than half (52.7%) of the participants strongly agreed that the training on EPI should be conducted at regular intervals. Moreover, 43.0% of the participants highly supported the notion that the eradication of diseases from a specific region is possible through EPI. Additionally, 57.5% of the participants strongly advocated for the fact that training in cold chain management is crucial for maintaining the efficacy of vaccines. A higher proportion of vaccinators (65.6%) strongly agreed about the importance of cold chain management in maintaining the efficacy of the vaccines. A significant statistical difference was also determined among some attitude-related variables between training and non-training participants (*p* < 0.05) (Table 3).

**Table 3:** Attitudes of vaccinators regarding EPI.

**Section 3 questions**	**Responses (*n* = 186)**	**Training (Yes; *n* = 79)**	**No training (No; *n* = 107)**	^*^p-value
Do you think the national immunization program contributed to a significant decrease in childhood morbidity and mortality rates?				**0.016**
SD	5 (2.7)	2 (2.5)	3 (2.8)	
D	4 (2.2)	0 (0.0)	4 (3.7)	
U	6 (3.2)	4 (5.1)	2 (1.8)	
A	65 (34.9)	19 (24.0)	46 (43.0)	
SA	106 (57.0)	54 (68.4)	52 (48.6)	
Do you think children who have missed any scheduled dose should be vaccinated afterward to complete the schedule according to their current age?				0.161
SD	3 (1.6)	1 (1.2)	2 (1.8)	
D	10 (5.4)	2 (2.5)	8 (7.5)	
U	17 (9.1)	6 (7.6)	11 (10.3)	
A	91 (48.9)	35 (44.3)	56 (52.3)	
SA	65 (34.9)	35 (44.3)	30 (27)	
Do you think training on EPI at regular intervals is necessary for healthcare workers?				**0.000**
SD	3 (1.6)	1 (1.2)	2 (1.8)	
D	7 (3.8)	3 (3.8)	4 (3.7)	
U	6 (3.2)	3 (3.8)	3 (2.8)	
A	72 (38.7)	16 (20.2)	56 (52.3)	
SA	98 (52.7)	56 (70.9)	42 (39.2)	
Do you think EPI can eradicate diseases from a specific region?				0.083
SD	3 (1.6)	1 (1.2)	2 (1.8)	
D	7 (3.8)	1 (1.2)	6 (5.6)	
U	26 (14.0)	14 (17.7)	12 (11.2)	
A	70 (37.6)	23 (29.1)	47 (43.9)	
SA	80 (43.0)	40 (50.6)	40 (37.4)	
Do you think immunization program can increase life expectancy of an individual?				0.217
SD	9 (4.8)	6 (7.6)	3 (2.8)	
D	24 (12.9)	10 (12.6)	14 (13.0)	
U	26 (14.0)	9 (11.4)	17 (15.9)	
A	63 (33.9)	22 (27.8)	41 (38.3)	
SA	64 (34.4)	32 (40.5)	32 (29.9)	
Do you think training in cold chain management is necessary to prevent the efficacy of vaccines?				**0.000**
SD	1 (0.5)	0 (0)	1 (0.9)	
D	3 (1.6)	0 (0)	3 (2.8)	
U	10 (5.4)	1 (1.2)	9 (8.4)	
A	65 (34.9)	15 (19.0)	50 (46.7)	
SA	107 (57.5)	63 (79.7)	44 (41.2)	
Do you think observation of symptoms and adverse effects after vaccination is necessary?				0.326
SD	4 (2.2)	2 (2.5)	2 (1.8)	
D	8 (4.3)	3 (3.8)	5 (4.6)	
U	17 (9.1)	6 (7.6)	11 (10.3)	
A	65 (34.9)	22 (27.8)	43 (40.1)	
SA	92 (49.5)	46 (58.2)	46 (43)	
Do you think cold chain management plays an important role in maintaining the potency of vaccines?				**0.035**
SD	1 (0.5)	0 (0)	1 (0.9)	
D	2 (1.1)	0 (0)	2 (1.8)	
U	8 (4.3)	1 (1.2)	7 (6.5)	
A	53 (28.5)	17 (21.5)	36 (33.6)	
SA	122 (65.6)	61 (77.2)	61 (57.0)	

SA, strongly agree; A, agree; U, uncertain; D, disagree; SD, strongly disagree; n, frequency; %, percentage. Bold values indicate the *p* < 0.05.

### Practices of vaccinators regarding vaccine storage and cold chain management

In the current study, participants showed good practices toward EPI, vaccine storage, and cold chain management. The majority (93.5%) of the participants checked the expiry of vaccines at regular intervals to maintain the first expiry first out (FEFO) in their healthcare setting. The majority of the participants (84.4%) used to record the temperature of the vaccine storage refrigerator twice a day. A statistically significant difference was measured between the practice of keeping OPV in a freezer and participants with training (*p* < 0.05). A vast majority of the participants (91.4%) used to dispose of needles and syringes in safety boxes. Moreover, most participants (69.4%) also tend to ensure open the vaccine refrigerators less than two times a day (Table 4).

**Table 4:** Practices of vaccinators regarding vaccine storage and cold chain management.

**Questions**	**Yes response**	**Training**		**No response**	**Training**		***p*-value**
	**Yes**	**Yes**	**No**	**No**	**Yes**	**No**	
	***n*** **(%)**	***n*** **(%)**	***n*** **(%)**	***n*** **(%)**	***n*** **(%)**	***n*** **(%)**	
Do you check the expiry of vaccines at regular intervals and maintain FEFO (first expiry first out) in your store?	174 (93.5)	77 (44.2)	97 (55.8)	12 (6.5%)	2 (16.7)	10 (83.3)	0.062
Do you record the temperature of the refrigerator two times daily?	157(84.4)	68 (43.3)	89 (56.7)	29(15.6%)	11 (37.9)	18 (62.1)	0.59
Do you keep OPV in the freezer?	141 (75.8)	55 (39)	86 (70)	45(24.2%)	24 (53.3)	21 (46.7)	0.090
Do you keep the refrigerator temperature at 2–8°C?	168 (90.3)	72 (42.9)	96 (57.1)	18(9.7%)	7 (38.9)	11 (61.1)	0.746
Do you discard multi-dose vaccine vials without preservatives after 6 h of opening?	147 (79.0)	67 (45.6)	80 (54.4)	39(21.0%)	12 (30.7)	27 (69.3)	0.096
Do you keep diluents in the refrigerator along with the vaccine at least 12–24 h before use?	129 (69.4)	54 (41.8)	75 (58.1)	57(30.6%)	25 (43.8)	32 (56.2)	0.799
Do you use safety boxes for collection and disposal of used syringes, needles, and other injection materials?	170 (91.4)	75 (44.1)	95 (55.9)	16(8.6%)	4 (25.0)	12 (75.0)	0.139
Do you maintain cold chain inventory on a regular basis?	164 (88.2)	68 (41.5)	96 (58.5)	22(11.8%)	11 (50.0)	11 (50.0)	0.477
Do you maintain the freezer temperature between −15°C and −25°C?	131 (70.4)	52 (39.7)	28 (21.3)	55(29.6%)	27 (49.1)	28 (50.9)	0.237
Do you have emergency cold chain management equipment (ice box) in case the refrigerator is not working?	172 (92.5)	73 (42.4)	99 (57.6)	14(7.5%)	6 (42.8)	8 (57.2)	0.976
Do you use reconstituted vaccines before 6 h?	128 (68.8)	56 (43.7)	72 (56.3)	58(31.2%)	23 (39.6)	35 (60.4)	0.601
Do you ensure that vaccine refrigerators are opened < 2 times a day?	129 (69.4)	56 (43.4)	73 (56.6)	57(30.6%)	23 (40.3)	34 (59.7)	0.697

The findings of the logistic regression measured a statistically significant difference (*p* = 0.000) between years of services and training and no training for the participants. However, there was no statistically significant difference in sex, education, or workplace with training vs. no training (Table 5).

**Table 5:** Logistic regression analysis between demographic variables and training vs. no training.

**Variable**	**Characteristics**	**Responses (*n* = 186)**	**Training**		**β**	**Standard error**	***p*-value**
Sex		***n*** **(%)**	**Yes (*****n*** **=** **79)**	**No (*****n*** **=** **107)**	−0.340	0.348	0.329
	Female	123 (66.1)	48 (39.0)	75 (61.0)			
	Male	63 (33.9)	31 (49.2)	32 (50.8)			
Educational level					−0.159	0.231	0.490
	Matric	14 (7.5)	9 (64.3)	5 (35.7)			
	Intermediate	29 (15.6)	14 (48.3)	15 (51.7)			
	Bachelor's	116 (62.4)	43 (37.1)	73 (62.9)			
	Master's	27 (14.5)	13 (48.1)	14 (51.9)			
Years of service (years)					0.624	0.123	**0.000**
	Less than 1	80 (43.0)	16 (20.0)	64 (80.0)			
	1–2	48 (25.8)	24 (50.0)	24 (50.0)			
	3–4	16 (8.6)	7 (43.7)	9 (56.3)			
	4–5	13 (7.0)	9 (69.2)	4 (30.8)			
	More than 5	29 (15.6)	23 (79.3)	6 (20.7)			
Workplace					−0.112	0.141	0.424
	Teaching academic hospital	26 (14.0)	8 (30.8)	18 (69.2)			
	Ministry of health	8 (4.3)	4 (50.0)	4 (50.0)			
	Public health hospital	60 (32.3)	41 (68.3)	19 (31.7)			
	Private hospital	69 (37.1)	21 (30.4)	48 (69.6)			
	Military hospital	23 (12.4)	5 (21.7)	18 (78.3)			

Bold values show significant factors.

## Discussion

The current study provided a new understanding of the KAP of Pakistani healthcare workers toward EPI, vaccines, and cold chain management. The results of this study revealed that most of the vaccinators had a moderate to poor level of knowledge regarding EPI. A recent study has reported that healthcare workers with higher levels of knowledge of vaccines had a positive attitude toward childhood vaccination regimens compared to those with less knowledge, indicating the importance of knowledge about childhood vaccination programs (26). Healthcare workers are considered the most trustworthy and reliable source of information about diseases that are preventable with vaccines (27). All healthcare workers, including vaccinators, play a major role in vaccination programs; hence, they should be equipped with the latest knowledge and scientific advancements to correctly communicate with patients and the general population (28). Doctors, nurses, and lady health workers regularly interact with the community, and vaccinators also need to know more about the cold chain. Therefore, all healthcare workers play a crucial role in building vaccine confidence among patients, which contributes to vaccine acceptance and vaccination behavior (29). Our findings suggested that vaccinators must be knowledgeable and understand the risks of developing vaccine-preventable infections or vaccinating an immunocompromised individual, which could result in disease transmission to the patient and other staff members.

Most of the vaccinators had a positive attitude and were largely in agreement with the importance of cold chain management in EPI. While a sizable majority recognized the beneficial effect of the program on reducing mortality and morbidity rates, a significant number of the participants also recognized the urgency of following up on missing vaccine doses and the significance of frequent training on EPI. Our results revealed a favorable attitude toward the effectiveness of EPI, demonstrating their dedication to adherence and improvements in the programs. However, there are also disagreements (10% reported uncertain, disagreed, and strongly disagreed) among the participants about the potential impact of EPI on the elimination of diseases and the enhancement of life expectancy. This shows that there is an urgent need to explain any nuances regarding the implications and efficacy of EPI through continued education among healthcare workers to improve their understanding of the overall effectiveness of the program. It has been reported that the positive attitude and knowledge of healthcare workers regarding the measles, mumps, and rubella (MMR) vaccine are critical to eliminating measles from Europe (30).

By overcoming the communication barriers among healthcare workers, it is possible to strengthen the immunization-related education among them, thereby enhancing the uptake of vaccines against specific diseases (31). Furthermore, there is broad agreement on the need for cold chain management to maintain the efficacy of vaccines. This shows that the attitude of healthcare workers can be critical in guaranteeing the effectiveness of EPI and improved management of the cold chain. A recent study conducted in Ghana in 2021 has also highlighted the importance of knowledge and attitude of healthcare workers and emphasized pivotal steps such as maintenance of the cold chain and its relation to retaining the potency and efficiency of vaccines; this, without any doubt, can boost the outcomes of the vaccine campaigns and minimize duplication of efforts (32). Therefore, all vaccinators should have a positive attitude and possess comprehensive knowledge about vaccines, in general, to help build vaccine confidence in the community and respond to any questions (33).

In this study, the data regarding general practices and routines of healthcare workers regarding the storage of vaccines and management of the cold chain indicated that healthcare workers did not follow the standard protocols of vaccine storage and cold chain management stringently. This is a very alarming situation, as the proper storage of vaccines is crucial for the success of the EPI and for maintaining stability and efficacy. Cold chain maintenance is strongly recommended for the oral polio vaccine (OPV) until its administration (34). In countries such as Pakistan, where poliovirus is still prevalent (35), the inability of the healthcare providers to properly store OPV and maintain the cold chain can seriously affect the vaccine stability, which can lead to an inadequate immune response against poliovirus that can lead to disease outbreaks. Hence, healthcare workers should strictly follow the standard procedures regarding the management of vaccine storage by preserving the cold chain to attain the maximum benefits from immunization programs. Pakistan, being a third world country, has limited resources, and land connections to various outreached areas are often difficult to access, especially during severe weather conditions, which can break the cold chain in many cases. Similarly, polio cases are also mostly common in these remote areas; therefore, training for reducing risks should be encouraged to generate more related knowledge and to ensure fruitful practices.

Another important aspect identified in this study was the lack of training related to EPI among vaccinators. A prior study revealed that participants who received training on EPI had a higher level of knowledge and vaccine-related information (33). It is recommended to provide various training approaches that may help vaccinators to increase their knowledge, skills, and competency in managing vaccination-related data (36). Therefore, periodic and compulsory training for all vaccinators on EPI activities, cold storage, vaccine cold chain management, the goal of EPI, and the resources available for the EPI program is required.

This study has some limitations. The sample size was small and included only the twin cities (Islamabad and Rawalpindi) located in a single geographical region; hence, the responses attained cannot be generalized to other regions of the country. The participants were chosen via convenience sampling, so it is possible that the sample does not truly represent the total population. A more diverse sample in future studies, including vaccinators from different regions, could provide a more comprehensive understanding. No causal interpretations can be derived from the study because it was a cross-sectional approach. Additionally, the current study makes use of self-reported data, which can be biased. Future research is required to correlate the KAP findings with actual immunization outcomes and also to plan and handle these issues. Despite these limitations, we state that our results are sound and will assist authorities in improving EPI activities in the future. This is the first study in Pakistan on vaccinators' KAP on EPI, vaccine cold storage, and cold chain management, which will serve as the baseline data for future research. The current study's findings suggest that all vaccinators should obtain EPI training to provide better public healthcare. To achieve this goal, hands-on training, emendations, refresher courses, and workshops for dealing with EPI must be initiated at the hospital and community levels.

## Conclusion

In the present study, most of the vaccinators had moderate to poor knowledge, positive attitudes, and good practices toward EPI, vaccine cold storage, and cold chain management. Lack of training among vaccinators on EPI was also observed. These findings have suggested that continuous training, education, and regular supervision of healthcare workers in EPI are important for maximum immunization effectiveness and coverage. Various strategies, including investing in resources and infrastructure to support the cold chain, ensuring the supply of vaccinations, and implementing specialized training programs to increase the abilities of healthcare providers, are crucial for better immunization programs and healthcare systems. Furthermore, future multi-center and regional-level qualitative research studies are required to gain a more comprehensive understanding of the KAP levels of different populations regarding EPI.

## Data availability statement

The original contributions presented in the study are included in the article/Supplementary material, further inquiries can be directed to the corresponding author.

## Ethics statement

This study was approved by the Ethical Committee of Yusra Institute of Pharmaceutical Sciences (Reference number: YIPS/4065/EC-17; Dated: 01/01/2021) . Informed consent both oral and written was obtained from all participants.

## Author contributions

SAz: Conceptualization, Data curation, Formal analysis, Investigation, Methodology, Software, Validation, Visualization, Writing—original draft, Writing—review & editing. LR: Conceptualization, Data curation, Formal analysis, Investigation, Methodology, Software, Validation, Visualization, Writing—original draft, Writing—review & editing. TI: Conceptualization, Investigation, Methodology, Validation, Visualization, Writing—original draft, Writing—review & editing. SAk: Conceptualization, Data curation, Methodology, Validation, Visualization, Writing—original draft, Writing—review & editing. KH: Conceptualization, Data curation, Validation, Visualization, Writing—original draft, Writing—review & editing. TS: Conceptualization, Data curation, Formal analysis, Methodology, Validation, Visualization, Writing—original draft, Writing—review & editing. AI: Conceptualization, Data curation, Formal analysis, Visualization, Writing—original draft, Writing—review & editing. NAn: Conceptualization, Data curation, Formal analysis, Validation, Visualization, Writing—original draft, Writing—review & editing. NM: Conceptualization, Data curation, Formal analysis, Validation, Visualization, Writing—original draft, Writing—review & editing. ZK: Conceptualization, Data curation, Formal analysis, Methodology, Validation, Visualization, Writing—original draft, Writing—review & editing. NAh: Conceptualization, Data curation, Formal analysis, Validation, Visualization, Writing—original draft, Writing—review & editing. HA: Conceptualization, Data curation, Investigation, Methodology, Project administration, Software, Supervision, Validation, Visualization, Writing—original draft, Writing—review & editing.
