# Supplementary material for: Knowledge, attitudes, and practices of vaccinators about expanded programs on immunization: a cross-sectional study
Source: Front Public Health. 2024 Mar 6;12:1366378. doi: 10.3389/fpubh.2024.1366378 (PMC10953913; doi:10.3389/fpubh.2024.1366378)
Supplement: Supplementary file 1 [file Data_Sheet_1.PDF]

Vaccinators' Knowledge, Attitude and Practices Regarding Expanded Program on Immunization

QUESTIONNAIRE

(English version)

SECTION # 1	DEMOGRAPHIC DATA:
Name	
Gender	Male : ☐ female: ☐ others: ☐
Age	
Educational status	Matric: ☐ Intermediate: ☐ Bachelor: ☐ Masters: ☐
Position	Vaccinator: ☐
Year/s of service in EPI	
Training on EPI	Yes : ☐ No: ☐
Any other vaccine related training	
Name of training	
Duration of training	
Work place	Teaching/Academic hospital ☐<br>Ministry of Health ☐<br>Public Health hospital ☐<br>Private hospital ☐<br>Others (specify)____________________

Section # 2 Knowledge of Vaccinators Regarding Expanded Programme on Immunization			
The rating scale is as follows (Put ✓ on the response that applies to you)			
**Vaccine name**	**Questions**	**Agree / yes**	**Disagree / No**
1. BCG	a. Can BCG be administered to HIV positive patient?		
	b. Is dose of BCG 0.5ml?		
	c. Can BCG be administered IM?		
	d. Is BCG vaccine given against T.B.?		
2. Polio vaccine	a. Do you ask for diarrhea symptoms of child before administration of polio vaccine?		
	b. Should OPV vaccine be repeated afterwards for children having severe diarrhea?		
	c. Can we adjust dose of OPV vaccine according to the weight of neonatal?		

	d. Do you think polio vaccine can cause polio disease to some individuals?		
	e. Is there any other route, except oral for administration of polio vaccine?		
3. DPT	a. Dose P in DTaP stands for pneumonia?		
	b. Can DPT be administered at time of birth?		
	c. Do you think DPT loses potency on freezing?		
4. Tetanus (maternal)	a. Can pregnant woman receive tetanus vaccine?		
	b. Do you think neonatal tetanus deaths can be prevented by maternal tetanus vaccination?		
	c. Is second dose of tetanus given to mother after four weeks?		
5. Hepatitis B	a. Can dose of pentavalent (DPT+HBV+Hib) be repeated if adverse effects are reported?		
	b. Can dose of hepatitis B vaccine be given through subcutaneous route?		
	c. Is first dose of Hepatitis B given within 24 hours of child birth?		
6. Hib	a. Is Hib vaccine used against influenza?		
	b. Can Hib be administered IV?		
7. MMR	a. Dose R in MMR stands for rabies?		
	b. Is MMR vaccine only be administered after 9 months?		

Questions	Agree / yes	Disagree / No
**8. Can live vaccines be given to immunocompromised individuals?**		
**9. Can IM vaccine dose be given more than 0.5 ml?**		
**10. Can live vaccines be given to immunocompromised individuals?**		
**11. Is necessary to administer multiple dose of same antigen to individual after four weeks of first dose?**		
**12. Do you check temperature before vaccination?**		

13. Is it recommended to delay vaccination in persons having high grade fever >39°C?		
14. Persons having chronic kidney, liver or heart disease can receive vaccination?		
15. Patients on medications (such as; antibiotics or corticosteroids) can receive vaccination?		

**Section # 3 Attitude of Vaccinators Regarding Expanded Programme on Immunization**					
	**Strongly agree**	**Agree**	**Neither agree nor disagree**	**Disagree**	**Strongly disagree**
1. Do you think the national immunization programme contributed in significant decrease in childhood morbidity and mortality?					
2. Do you think children who have missed any scheduled dose should be vaccinated afterward to complete the schedule according to their current age?					
3. Do you think training on EPI on regular interval is necessary for healthcare workers?					
4. Do you think EPI can eradicate diseases from a specific region?					
5. Do you think immunization program can increase life expectancy of an individual?					
6. Do you think training in cold chain management is necessary to prevent the efficacy of vaccines?					
7. Do you think observation of symptoms and adverse effects after vaccination is necessary?					
8. Do you think cold chain management plays an important role in maintaining potency of vaccines?					

**Section # 4 Practices of Vaccinators Regarding Expanded Programme on Immunization**		
Questions	Yes	No
1. Do you check expiry of vaccines on regular interval, and maintain FEFO in your store?		
2. Do you record temperature of refrigerator two times daily?		

3. Do you keep OPV in freezer?		
4. Do you keep refrigerator temperature at 2 to 8°C?		
5. Do you discard multi-dose vaccine vial after 28 days?		
6. Do you discard multi-dose vaccine vial without preservative after 6 hours of opening?		
7. Do you keep diluents in refrigerator with vaccine at least 12-24 hours before use?		
8. Do you maintain stock registers of vaccines on regular interval?		
9. Do you use safety boxes for collection and disposal of used syringes, needles and other injection materials?		
10. Do you maintain cold chain inventory on regular basis?		
11. Do you update vaccination card of individual on regular interval?		
12. Do you maintain freezer temperature at -15°C to -25°C?		
13. Do you have emergency cold chain management equipment (ice box) in case the refrigerator is not working?		
14. Do you avoid placing food and drinks with vaccine, as it can affect vaccine potency?		
15. Do you place “open when needed label on the door of every refrigerator?		
16. Do you use reconstituted vaccines before 6 hours?		
17. Do you ensure that vaccine refrigerators are opened < 2 times a day?		

(Urdu version)

حفاظتی ٹیکوں سے متعلق توسیعی پروگرام کے حوالے سے ویکسینیٹر کا علم، رویہ اور طرز عمل

سوالنامہ

ممتاز!

ہم سب جانتے ہیں کہ ویکسین عالمی سطح پر متعدی وزیروں کو بہت کم کر دیا اور ان کے مقابلے کو ڈرامائی پر کم کر دیا جو کبھی بڑے پیمانے پر اور عام مہلک ہوتا ہے، ہم اس تحقیق میں۔ جو بھی تعاون کر سکتے ہیں اس کی تعریف کر سکتے ہیں۔ آدمی یہ بات 43 بند سوالات پر مشتمل ہے اور اسے ایک فرد کو اوسطاً 10 منٹ لگتے ہیں۔ ہم یقینی طور پر یقینی ہیں کہ آپ کا جواب محفوظ رکھا جائے گا اور ہمارے پاس محفوظ ہے۔

ڈیموگرافک ڈیٹا:	سیکشن #1
نام	
صنف	مرد: عورت: دیگر: ☐ ☐ ☐
عمر	
تعلیمی حیثیت	میٹرک: انٹرمیڈیٹ: بیچلر ماسٹرز:
پوزیشن	ویکسینیٹر ☐
EPI میں سروس کے سال	
ای پی آئی پر تربیت	ہاں نہیں: ☐ ☐
ویکسین سے متعلق کوئی دوسری تربیت	
تربیت کا نام	
تربیت کا دورانیہ	
کام کی جگہ	تدریسی/تعلیمی ہسپتال ☐<br>وزارت صحت ☐<br>پبلک ہیلتھ ہسپتال ☐<br>پرائیویٹ ہسپتال ☐<br>دوسرے)وضاحت کریں(____________________

**حفاظتی ٹیکوں سے متعلق توسیعی پروگرام کے حوالے سے ویکسینیٹر کا علم** دفعہ نمبر 2				
درجہ بندی کا پیمانہ درج ذیل ہے)جو جواب آپ پر لاگو ہوتا ہے اس پر ✓ لگائیں(				
**ویکسین کا نام**	**سوالات**	**متفق /ہاں**	**متفق/نہیں**	**/ ہو سکتا ہے مجھے نہیں معلوم**
16. بی سی جی	e. کیا بی سی جی ایچ آئی وی پازیٹو مریض کو دیا جا سکتا ہے؟			
	f. کیا BCG 0.5 mlکی خوراک ہے؟			
	g. کیا BCG کو IM دیا جا سکتا ہے؟			
	h. ویکسین BCG کیا ٹی بی کے خلاف دی جاتی ہے؟			
17. پولیو ویکسین	f. کیا آپ پولیو ویکسین پلانے سے پہلے بچے میں اسہال کی علامات پوچھتے ہیں؟			

	.g کیا شدید اسہال والے بچوں کے لیے ویکسین کو بعد میں دہرایا جانا OPV چاہیے؟			
	.h کیا ہم نوزائیدہ کے وزن کے مطابق ویکسین کی خوراک کو ایڈجسٹ OPV کر سکتے ہیں؟			
	.i کیا آپ کے خیال میں پولیو ویکسین کچھ افراد کو پولیو کی بیماری کا باعث بن سکتی ہے؟			
	.j کیا پولیو ویکسین کے انتظام کے لیے زبانی کے علاوہ کوئی دوسرا راستہ ہے؟			
18. ڈی پی ٹی	.d کا مطلب ہے۔ P میں خوراک DTaP نمونیا؟			
	.e کیا پیدائش کے وقت ڈی پی ٹی کا انتظام کیا جا سکتا ہے؟			
	.f کیا آپ کو لگتا ہے کہ ڈی پی ٹی جمنے پر طاقت کھو دیتا ہے؟			
19. تشنج (زچگی)	.d کیا حاملہ عورت تشنج کی ویکسین حاصل کر سکتی ہے؟			
	.e کیا آپ کے خیال میں نوزائیدہ تشنج سے ہونے والی اموات کو زچگی کے ٹیٹنس کی ویکسینیشن سے روکا جا سکتا ہے؟			
	.f کیا تشنج کی دوسری خوراک چار ہفتوں کے بعد ماں کو دی جاتی ہے؟			
20. کالا یرقان	.d اگر منفی اثرات کی اطلاع دی جائے تو (DPT+HBV+Hib) کیا پینٹا ویلنٹ کی خوراک کو دہرایا جا سکتا ہے؟			
	.e کیا ہیپاٹائٹس بی ویکسین کی خوراک ذیلی راستے سے دی جا سکتی ہے؟			
	.f کیا ہیپاٹائٹس بی کی پہلی خوراک بچے کی پیدائش کے 24 گھنٹے کے اندر دی جاتی ہے؟			
21. حب	.c ویکسین انفلوئنزا کے خلاف Hib کیا استعمال کی جاتی ہے؟			
	.d کیا جا سکتا ہے؟ IV کا انتظام Hib کیا			
22.ایم ایم آر	.c کا مطلب ریبیز R میں خوراک MMR ہے؟			
	.d ویکسین صرف 9 ماہ کے MMR کیا بعد لگائی جاتی ہے؟			

سوالات	متفق /ہاں	متفق/نہیں	ہو سکتا ہے / مجھے نہیں معلوم
23. کیا امیونوکمپرومائزڈ افراد کو زندہ ویکسین دی جا سکتی ہیں؟			
24. ویکسین کی خوراک 0.5 ملی لیٹر سے زیادہ IM کیا دی جا سکتی ہے؟			
25. کیا امیونوکمپرومائزڈ افراد کو زندہ ویکسین دی جا سکتی ہیں؟			

26. کیا پہلی خوراک کے چار ہفتوں کے بعد فرد کو ایک ہی اینٹیجن کی متعدد خوراکیں دینا ضروری ہے؟			
27. کیا آپ ویکسینیشن سے پہلے درجہ حرارت چیک کرتے ہیں؟			
28. والے افراد میں C°کیا اعلی درجے کا بخار>39 ویکسینیشن میں تاخیر کی سفارش کی جاتی ہے؟			
29. دائمی گردے، جگر یا دل کی بیماری والے افراد ویکسینیشن حاصل کر سکتے ہیں؟			
30. ادویات لینے والے مریض)جیسے؛ اینٹی بائیوٹکس یا کورٹیکوسٹیرائڈز (ویکسینیشن حاصل کر سکتے ہیں؟			

**سیکشن نمبر 3 حفاظتی ٹیکوں کے توسیعی پروگرام کے حوالے سے ویکسینیٹر کا رویہ**					
	بہت زیادہ اتفاق	متفق	نہ متفق نہ اختلاف	اختلاف کرنا	بہت زیادہ اختلاف
9. کیا آپ کو لگتا ہے کہ قومی حفاظتی ٹیکوں کے پروگرام نے بچپن کی بیماری اور اموات میں نمایاں کمی کی ہے؟					
10. کیا آپ کو لگتا ہے کہ جن بچوں نے کوئی مقررہ خوراک چھوٹ دی ہے ان کو ان کی موجودہ عمر کے مطابق شیڈول مکمل کرنے کے لیے بعد میں ٹیکہ لگایا جانا چاہیے؟					
11. کیا آپ کو لگتا ہے کہ صحت کی دیکھ بھال کرنے کی EPI والے کارکنوں کے لیے باقاعدہ وقفہ پر تربیت ضروری ہے؟					
12. کسی مخصوص علاقے EPI کیا آپ کے خیال میں سے بیماریوں کو ختم کر سکتا ہے؟					
13. کیا آپ کے خیال میں حفاظتی ٹیکوں کا پروگرام کسی فرد کی متوقع عمر میں اضافہ کر سکتا ہے؟					
14. کیا آپ کو لگتا ہے کہ ویکسین کی افادیت کو روکنے کے لیے کولڈ چین مینجمنٹ کی تربیت ضروری ہے؟					
15. کیا آپ کے خیال میں ویکسینیشن کے بعد علامات اور منفی اثرات کا مشاہدہ ضروری ہے؟					
16. کیا آپ کے خیال میں کولڈ چین مینجمنٹ ویکسین کی طاقت کو برقرار رکھنے میں اہم کردار ادا کرتی ہے؟					

**سیکشن #4 حفاظتی ٹیکوں پر توسیعی پروگرام کے حوالے سے ویکسینیٹر کے طرز عمل**		
سوالات	جی ہاں	نہیں
18. کیا آپ باقاعدہ وقفہ پر ویکسین کی میعاد ختم ہونے کی جانچ کرتے ہیں، اور کو برقرار رکھتے ہیں؟ FEFO اپنے اسٹور میں		
19. کیا آپ روزانہ دو بار فریج کا درجہ حرارت ریکارڈ کرتے ہیں؟		
20. کو فریزر میں رکھتے ہیں؟ OPV کیا آپ		
21. کیا آپ ریفریجریٹر کا درجہ حرارت 2 سے 8 ڈگری سینٹی گریڈ پر رکھتے ہیں؟		
22. کیا آپ 28 دنوں کے بعد ملٹی ڈوز ویکسین کی شیشی کو ضائع کر دیتے ہیں؟		
23. کیا آپ ملٹی ڈوز ویکسین کی شیشی کو کھولنے کے 6 گھنٹے بعد پریزرویٹیو کے بغیر ضائع کر دیتے ہیں؟		
24. کیا آپ استعمال سے کم از کم 12 سے 24 گھنٹے پہلے ویکسین کے ساتھ ریفریجریٹر میں ڈلوئنٹس رکھتے ہیں؟		

25. کیا آپ باقاعدہ وقفہ پر ویکسین کے اسٹاک رجسٹر کو برقرار رکھتے ہیں؟		
26. کیا آپ استعمال شدہ سرنجوں، سوئیوں اور انجیکشن کے دیگر مواد کو جمع کرنے اور ٹھکانے لگانے کے لیے حفاظتی بکس استعمال کرتے ہیں؟		
27. کیا آپ کولڈ چین انوینٹری کو باقاعدگی سے برقرار رکھتے ہیں؟		
28. کیا آپ باقاعدہ وقفہ پر فرد کے ویکسی نیشن کارڈ کو اپ ڈیٹ کرتے ہیں؟		
29. کیا آپ فریزر کا درجہ حرارت -15 ^{o}C سے -25 ^{o}C پر برقرار رکھتے ہیں؟		
30. ریفریجریٹر کام نہ کرنے کی صورت میں کیا آپ کے پاس ایمرجنسی کولڈ چین مینجمنٹ کا سامان)آئس باکس (ہے؟		
31. ،کیا آپ خوراک اور مشروبات کو ویکسین کے ساتھ رکھنے سے گریز کرتے ہیں کیونکہ یہ ویکسین کی طاقت کو متاثر کر سکتا ہے؟		
32. کیا آپ ہر فریج کے دروازے پر "ضرورت کے وقت کھلا "کا لیبل لگاتے ہیں؟		
33. کیا آپ دوبارہ تشکیل شدہ ویکسین 6 گھنٹے سے پہلے استعمال کرتے ہیں؟		
34. کیا آپ اس بات کو یقینی بناتے ہیں کہ ویکسین کے ریفریجریٹرز دن میں >2 بار کھولے جاتے ہیں؟		
